# TP53 and LRP1B Co-Wild Predicts Improved Survival for Patients with LUSC Receiving Anti-PD-L1 Immunotherapy

**DOI:** 10.3390/cancers14143382

**Published:** 2022-07-12

**Authors:** Jiangyong Yu, Zaiwen Fan, Zhipeng Zhou, Ping Zhang, Jing Bai, Xu Li, Min Tang, Nannan Fan, Xiaonan Wu, Xin Nie, Xiaoyan Chen, Di Ma, Xi Chen, Liang Cui, Xuefeng Xia, Ling Yang, Xin Yi, Lin Li

**Affiliations:** 1 Department of Medical Oncology, Beijing Hospital, National Center of Gerontology, Institute of Geriatric Medicine, Chinese Academy of Medical Sciences, Beijing 100730, China; yujiangyong2011@163.com (J.Y.); zhangping990721@126.com (P.Z.); 13810865876@139.com (X.L.); tangmin3911@bjhmoh.cn (M.T.); nkfan1986@163.com (N.F.); wuxn2011@126.com (X.W.); evening841103@163.com (X.N.); chenxiaoyan0206@163.com (X.C.); mdyxy08@163.com (D.M.); chenxi4834@bjhmoh.cn (X.C.); 2 Department of Medical Oncology, Air Force Medical Center, PLA, Beijing 100142, China; kzzaiwenfan@163.com; 3 Geneplus-Beijing Institute, Beijing 102206, China; zhouzhp@geneplus.org.cn (Z.Z.); baijing@geneplus.org.cn (J.B.); cuiliang@geneplus.org.cn (L.C.); xiaxf@geneplus.org.cn (X.X.); yangl@geneplus.org.cn (L.Y.); yix@geneplus.org.cn (X.Y.)

**Keywords:** LUSC, immunotherapy, *TP53*, *LRP1B*, biomarker

## Abstract

**Simple Summary:**

Immune checkpoint inhibitors (ICIs) changed the standard care of patients with lung squamous cell carcinoma (LUSC). It is an urgent need to precisely predict the population’s response to ICIs. The aim of the study was to explore novel biomarkers for LUSC immunotherapy and the potential mechanism. Five hundred and twenty-five Chinese patients with LUSC who underwent targeted sequencing were involved, and *TP53* and *LRP1B* were the genes most frequently correlated to tumor mutational burden (TMB). Further analysis demonstrated that *TP53*/*LRP1B* co-wild was associated with improved survival of immunotherapy. Compared with those with *TP53*/*LRP1B* mutant, patients with *TP53*/*LRP1B* co-wild had lower TMB and chromosome instability, while stronger cytotoxic immune cell infiltrations, which might be the reason for the better clinical outcomes of immunotherapy. These results indicated that *TP53*/*LRP1B* co-wild might be regarded as a potential biomarker for guiding anti-PD-L1 immunotherapy in LUSC.

**Abstract:**

Immunotherapy brought long-term benefits for partial patients with lung squamous cell carcinoma (LUSC). The predictor of anti-PD-L1 therapy was controversial and limited in LUSC. We aimed to explore novel biomarker for LUSC immunotherapy and the potential mechanism. Five hundred and twenty-five Chinese patients (Geneplus cohort) with LUSC underwent targeted sequencing and were involved to explore the genomic profiling. *TP53* and *LRP1B* were the most frequently recurrent genes and correlated to higher tumor mutational burden (TMB). We observed that LUSC patients with *TP53* and *LRP1B* co-wild (co-wild type) were associated with better survival of anti-PD-L1 therapy compared with *TP53* mutant or *LRP1B* mutant (mutant type) in POPAR/OAK cohort. Copy-number variation (CNV) and whole genome doubling (WGD) data from TCGA LUSC cohort were obtained to assess the CNV events. There were fewer CNV alterations and lower chromosome instability in patients with *TP53*/*LRP1B* co-wild compared with those with *TP53*/*LRP1B* mutant. RNA expression data from the TCGA LUSC cohort were collected to explore the differences in RNA expression and tumor immune microenvironment (TIME) between mutant and co-wild groups. The *TP53*/*LRP1B* co-wild type had a significantly increased proportion of multiple tumor-infiltrating lymphocytes (TILs), including activated CD8 T cell, activated dendritic cell (DC), and effector memory CD8 T cell. Immune-related gene sets including checkpoint, chemokine, immunostimulatory, MHC and receptors were enriched in the co-wild type. In conclusion, *TP53*/*LRP1B* co-wild LUSC conferred an elevated response rate in anti-PD-L1 therapy and improved survival, which was associated with a chromosome-stable phenotype and an activated immune microenvironment.

## 1. Introduction

Lung squamous cell carcinoma (LUSC) is one of the most common histological subtypes in lung cancer, accounting for about 30% of non-small cell lung cancer (NSCLC) patients [1]. Several studies have demonstrated that most LUSC patients are currently or were previously heavy smokers, which results in highly complex genome complexity and enhancive overall mutation load [2,3]. LUSC has few potential driving gene targets and lacks targeted drug therapy. Conventional platinum-based combination chemotherapy has always remained the first-line therapy for advanced LUSC, but the survival benefit is limited [1,4,5]. In recent years, the rise of immune checkpoint inhibitors (ICIs) has changed the treatment paradigm of LUSC [6,7,8,9,10]. Lee J.S. et al. revealed that anti-PD-1/PD-L1 therapy could provide significant survival benefits and a high objective response rate for LUSC patients [11]. Moreover, several immunomonotherapy or immunochemotherapy studies such as RATIONALE-307 [12], KEYNOTE-407 [8], and CheckMate-017 [6] showed that ICIs improved the survival of advanced LUSC and provided new alternatives for first/second-line treatment.

Unfortunately, only a minority of LUSC patients respond to current ICIs [9], and thus identifying biomarkers to determine the potentially beneficial effect on the population has become an important topic of immunotherapy. Positive PD-L1 expression and high tumor mutation burden (TMB) were considered to predict the response to immunotherapy in NSCLC [13,14], but there is a subset of LUSC patients with PD-L1 expression <1% who benefit from ICIs, suggesting that PD-L1 IHC staining alone is not perfect enough to identify all potential immunotherapy responses in the population [6]. In addition, PD-L1 as a biomarker was limited by the multitude of PD-L1 antibodies, assays, scoring systems, and thresholds for positivity currently used [15]. CheckMate-227 [14] showed that nonsquamous histologic type had longer progression-free survival with the combination of nivolumab plus ipilimumab than squamous type in NSCLC patients with a high TMB. A recent study revealed that TMB as a biomarker for predicting the efficacy of immunotherapy was not applicable for metastatic LUSC [16]. Collectively, PD-L1 and TMB were very limited in predicting LUSC immunotherapy beneficiaries. Therefore, the development of more effective biomarkers has become an imperious demand for LUSC immunotherapy.

Herein, in order to explore novel biomarkers, we investigated the genomic landscape of 525 LUSC patients and its correlation with TMB and PD-L1 expression. We found that *TP53* and *LRP1B* genes were most associated with TMB, and *TP53* was associated with PD-L1 expression. Further analysis revealed that the *TP53*/*LRP1B* co-wild LUSC had a lower level of TMB and chromosome instability (CIN), and stronger antigen presentation ability and cytotoxic immune cell infiltrations, which significantly prolonged progression-free survival (PFS) and overall survival (OS) in LUSC patients treated with mono-ICI treatment. These results indicated that *TP53*/*LRP1B* co-wild might be regarded as a potential predictor for guiding anti-PD-L1 immunotherapy in LUSC.

## 2. Materials and Methods

### 2.1. Patients and Tumor Samples

Between January 2018 to October 2020, a total of 525 patients with LUSC underwent a next-generation sequencing (NGS) assay which contains 1021 genes (Supplementary Table S1) in the Geneplus-Beijing Institute, Beijing, China (Geneplus cohort, Supplementary Table S2). Written informed consent was obtained from all patients. Participants’ characteristics (i.e., age, sex, smoking status) were collected during initial enrollment (Supplementary Table S3). The study was an observational, non-interventional study and was approved by the ethical committee at Beijing Hospital.

### 2.2. NGS Sequencing and Bioinformatics Analysis

DNA extraction and library construction were performed as previously described [17]. Briefly, genomic DNA was extracted from microdissected tissue sample and concentrations were measured using a Qubit fluorometer and the Qubit dsDNA HS Assay Kit (Invitrogen, Carlsbad, CA, USA). Extracted DNA was sheared to 300 bp fragments with a Covaris S2 ultrasonicator (Covaris, Woburn, MA, USA). Fragmented DNA was added to Illumina-indexed adapters for library construction using the KAPA Library Preparation Kit (Kapa Biosystems, Wilmington, MA, USA). Sequencing libraries were hybridized to custom-designed probes (NimbleGen, Roche) of biotinylated oligonucleotides. Massively parallel sequencing was performed using HiSeq3000 Sequencing System with 2 × 100 bp paired-end reads. Reads were aligned to the reference sequence b37 edition from the Human Genome Reference Consortium using BWA (version 0.5.9, Broad Institute, Cambridge, MA, USA) [18]. Hybridization capture sequencing revealed a mean effective depth of coverage over 1000× in samples. Single nucleotide variants (SNVs) were called using MuTect (version 1.1.4) and NChot [19,20]. Small insertions and deletions (Indels) were determined by GATK [21].

### 2.3. IHC for PD-L1

PD-L1 expression levels were evaluated in LUSCs tissue samples by IHC staining by means of the PD-L1 IHC 22C3 pharmDx assay (DAKO). The detectable sample needs to meet the criteria for more than 100 living tumor cells. The immunohistochemistry results were quantized as tumor proportion score (TPS) based on the degree and intensity of cell membrane staining.

### 2.4. LUSC Data Collect

We collected 125 LUSC patients from the POPLAR [22] and OAK [10] cohorts who received anti-PD-L1 immunotherapy (Atezolizumab) to explore the clinical outcome of the anti-PD-L1 immunotherapy between groups with different genotypes. Somatic mutation data, patient characteristics, and clinical data were obtained from a previous study [23]. We obtained matched tumor-normal whole-exome sequencing (WES) data (469 available), mRNA expression data (466 available), and copy-number variation (CNV) data (469 available) of 469 LUSC patients in the TCGA database from cBioPortal (https://www.cbioportal.org/ (accessed on 1 September 2020)) to analyze the copy number variations, differentially expressed gene (DEG), and tumor immune microenvironment (TIME).

### 2.5. TMB

For the Geneplus cohort, the TMB was calculated as the number of nonsynonymous SNVs divided by the length of the panel-covered genomic region (1.09 Mb). For the POPLAR [22] and OAK [10] cohorts, the blood-based TMB (bTMB) was evaluated by somatic nonsynonymous SNVs and indels detected in coding regions of blood samples using a companion diagnostic assay (FoundationOne). For the TCGA cohort, TMB was defined as the number of nonsynonymous SNVs and indels in the examined coding regions with the variated allele frequency (VAF) ≥1% in tumor tissues.

### 2.6. Oncogenic Signaling Pathway Analysis

Somatic mutations were classified into ten oncogenic signaling pathways, including Cell cycle, HIPPO, MYC, NOTCH, NRF2, PI3K, RTK-RAS, TGF-beta, TP53, and WNT according to previous studies [24]. Fisher’s exact test was used to reveal the potential difference in oncogenic mechanisms between co-wild and mutant LUSC.

### 2.7. CNV Analysis

Focal copy number alterations in co-wild and mutant LUSC were identified by segmented data in TCGA cohort using GISTIC2.0 [25]. The whole genome doubling (WGD) and ploidy data were determined by a previous study [26]. The genomic instability index (GII) was calculated as the sum length of copy number alterations region divided by the total genome length.

### 2.8. Differentially Expressed Genes Analysis and Pathway Enrichment

Differentially expressed genes (DEG) analysis was performed using the R package “DEseq2”. The RSEM (RNAseq by expectation-maximization) values were rounded as input data. DEGs were identified with false discovery rate (FDR) < 0.05 and |log2FoldChange| > 1. DEGs pathway enrichment analysis was performed using the R package “clusterProfilier” with Gene Ontology (GO) database. The significant GO terms in each group were identified with a cutoff of FDR < 0.05.

### 2.9. GSEA

The mRNA expression data was transformed by log2(RSEM + 1) for gene set enrichment analysis (GSEA). Hallmark and immune-related gene sets were collected from previous studies as reference gene sets [27,28]. JavaGSEA 4.0 Desktop Application was used to identify significantly altered gene sets (FDR < 0.10) between co-wild and mutant LUSC.

### 2.10. Immune Microenvironment Assessment

The mRNA expression which was quantified by RSEM was transformed by log2(RSEM + 1) for TIME analysis. The single sample gene set enrichment analysis (ssGSEA) [29] was performed to quantify the relative infiltration of 28 immune cell types in the tumor immune microenvironment. Feature genes for each immune cell type were obtained from a previous study [29]. T cell-inflamed gene expression profile score (GEP-score) [30] was calculated according to a weighted mean of 18 immune inflammatory related genes including *CD274*, *CD276*, *CCL5*, *CD27*, *CD8A*, *CMKLR1*, *CXCL9*, *CXCR6*, *IDO1*, *LAG3*, *NKG7*, *PDCD1LG2*, *PSMB10*, *HLA-DQA1*, *HLA-DRB1*, *HLAE*, *STAT1*, and *TIGIT*. The antigen presentation and chemokine related gene were collected from previous studies [31,32]. FDR < 0.05 was considered statistically significant.

### 2.11. Statistical Analysis

Differences among the groups with normal distribution and equal variance were analyzed using the independent sample t-test. Comparisons with different variance were analyzed using the Wilcoxon test. The *p* values were obtained using the log-rank test and the Kaplan–Meier method for the estimation of PFS and OS. A two-sided *p* value < 0.05 was considered statistically significant. All statistical analyses were performed using R-3.6.0.

## 3. Results

### 3.1. Comprehensive Genomic Profiles of Patients with LUSC

**Figure 1:**
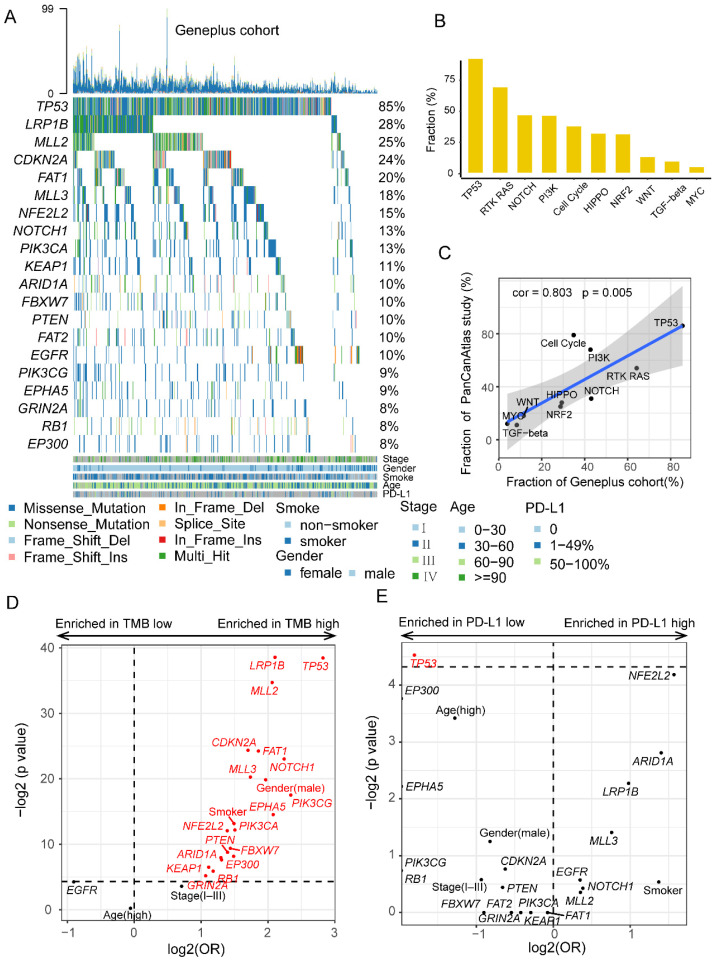
The mutational characteristics of patients with LUSC in Geneplus cohort. (**A**) The frequently recurrent genes in 525 LUSC patients. (**B**) The mutation frequency of ten carcinogenic signaling pathways in the Geneplus cohort. (**C**) The correlation between the mutation frequency of ten carcinogenic signaling pathways in the Geneplus cohort and PanCanAtlas study. (**D**) The clinical and mutational factors associated with TMB. (**E**) The clinical and mutational factors associated with PD-L1 expression.

Five hundred and twenty-five LUSC patients were enrolled in this study with median age of 64 years old (range 23–85). Four hundred and fifty-six (87%, 456/525) patients were male and two hundred and thirty-one (44%, 231/525) were smokers. Of all patients, 23% were in the metastatic stage (Stage IV, 121/525), 18% were in the localized or regional stages (Stage I–III, 92/525), and 59% (312/525) lacked detailed staging information. Clinical characteristics of the 525 patients are summarized in Supplementary Table S4. We examined the recurrently altered genes in this cohort (Figure 1A). *TP53* (85%), *LRP1B* (28%), *MLL2* (25%), *CDKN2A* (24%), and *FAT1* (20%) were the most frequent genes (Figure 1A). The percentage of *EGFR* (10%) mutation was higher than those in previous studies [33]. The median mutation load was 10 (range 0.96–95.04). We examined 10 canonical signaling pathways in this cohort and found TP53 (86%) and RTK-RAS (64%) pathways had a high mutation rate (Figure 1B) and the results showed a significant correlation with the PanCanAtlas study [24] (Figure 1C). PD-L1 expression data were obtained in 126 patients, including 45 (36%) patients with negative PD-L1 expression, 60 (48%) patients with expression level between 1–49%, and 21 (17%) patients with expression level between 50–100%.

Next, we examined which mutations or clinical factors were associated with TMB and PD-L1 expression (Figure 1D,E). The top 20 mutational genes, age, sex, and smoker status were involved in this analysis. We observed that many genes were associated with high TMB (≥15 muts/Mb), the most notable being *TP53* and *LRP1B* mutations (Figure 1D). In addition, we divided the data into four groups according to the status of those two genes and found the *TP53*/*LRP1B* co-mutation group was associated with the highest TMB, while the *TP53*/*LRP1B* co-wild group was associated with the lowest TMB (Supplementary Figure S1A). Similar results were obtained using the TCGA and POPLAR/OAK cohorts (Supplementary Figure S1B,C). For PD-L1 expression, only *TP53* mutation was associated with low PD-L1 expression (Figure 1E).

We also investigated the genomic landscape in another cohort of LUSC patients involved in the clinical trial of POPLAR/OAK (as shown in Figure 3A). *TP53* (64%) and *LRP1B* (31%) were the most frequently mutated genes, and 10 canonical signaling pathways enriched from the recurrent genes were significantly correlated with the PanCanAtlas study and the Geneplus cohort (Supplementary Figure S2A–C). It was significantly correlated between *TP53* and *LRP1B* mutations and TMB-H (Supplementary Figure S2D).

### 3.2. TP53/LRP1B Co-Wild LUSC Had an Improved Outcome in Receiving Anti-PD-L1 Immunotherapy

One hundred and twenty-five LUSC patients from the POPLAR/OAK cohort underwent anti-PD-L1 immunotherapy and were involved to explore the relationship between genomic alteration and clinical outcome. We evaluated whether bTMB was a potential efficacy predictor or prognosis factor for LUSC patients. There were no significant differences between bTMB-L and bTMB-H groups in PFS, OS and DCR, whether the upper quartile bTMB (16 mutations) or a median bTMB (10 mutations) was used as a cutoff (Supplementary Figure S3A–F).

**Figure 2:**
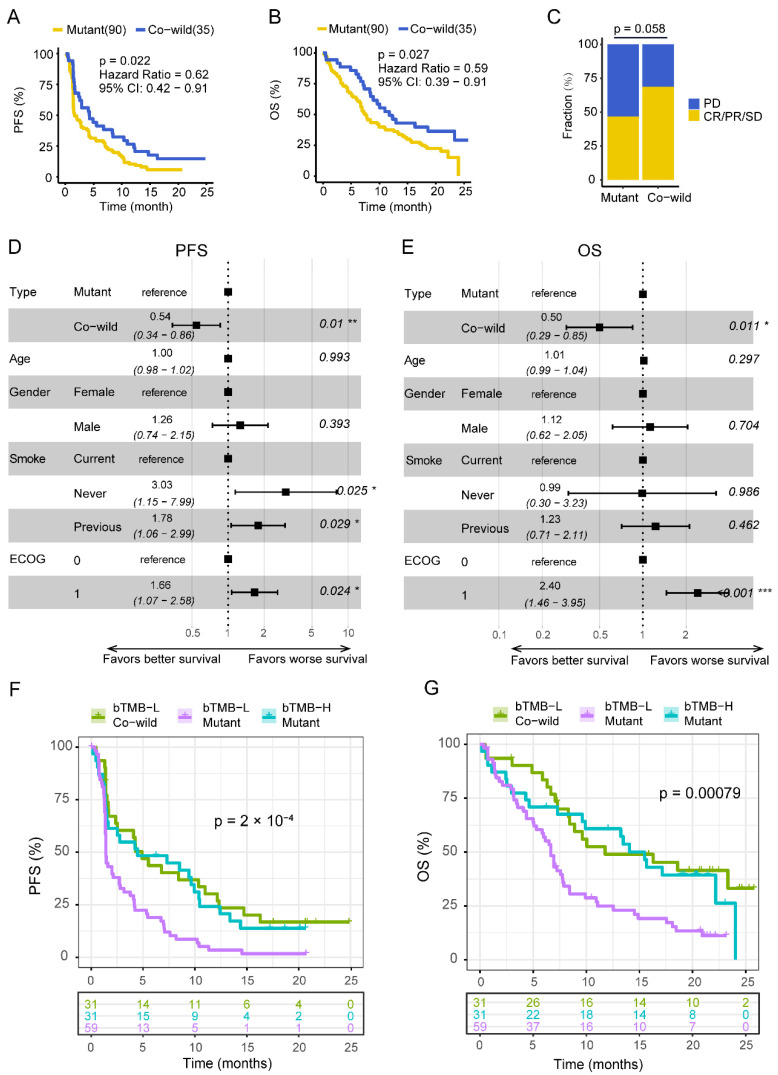
*TP53* and *LRP1B* co-wild was associated with better immunotherapy outcomes. (**A**,**B**) The results of survival analysis on PFS and OS between *TP53*/*LRP1B* mutant and co-wild LUSC in POPLAR/OAK cohort. (**C**) The differences of DCR between *TP53/LRP1B* mutant and co-wild LUSC in POPLAR/OAK cohort. (**D**,**E**) Multivariate cox regression analysis of PFS and OS, respectively. * *p* < 0.05, ** *p* < 0.01 and *** *p* < 0.001. (**F**,**G**) The results of survival analysis on PFS and OS among bTMB-H/mutant, bTMB-L/mutant, and bTMB-L/co-wild groups. PFS, progression-free survival; OS, overall survival; DCR, disease control rate; TMB-L, tumor mutational burden-low; TMB-H, tumor mutational burden-high.

Next, we focused on *TP53* and *LRP1B* genes. One hundred and twenty-five patients with LUSC were divided into four groups (A: *TP53*^mut^ and *LRP1B*^mut^, B: *TP53*^mut^ and *LRP1B*^wild^, C: *TP53*^wild^ and *LRP1B*^mut^, D: *TP53*^wild^ and *LRP1B*^wild^). Group D had prolonged FPS compared with the other three groups (A: 1.5 months, B: 1.6 months, C: 2.1 months, D: 4.2 months, *p* = 0.047) (Supplementary Figure S4A). The trend was similar in OS although the difference was not significant (A: 5.3 months, B: 7.8 months, C: 5.6 months, D: 11.0 months, *p* = 0.16) (Supplementary Figure S4B). Group D also had a higher disease control rate (DCR) (A: 50%, B: 46%, C: 44%, D: 69%) (Supplementary Figure S4C). We divided patients into *TP53*/*LRP1B* co-wild and mutant type (*LRP1B* mutant and/or *TP53* mutant) (Table 1, Supplementary Figure S5). Patients with *TP53*/*LRP1B* co-wild had a significantly prolonged PFS (4.2 vs. 1.6 months, HR = 0.62, *p* = 0.02) and OS (11.0 vs. 7.2 months, HR = 0.59, *p* = 0.03) as well as a marginally higher DCR (68.9% vs. 46.8%, *p* = 0.058) (Figure 2A–C). Multivariate cox regression analysis showed that *TP53*/*LRP1B* co-wild was associated with better clinical outcomes independent of age, sex, smoking status, and Eastern Cooperative Oncology Group (ECOG) score (Figure 2D,E).

Both bTMB and *TP53*/*LRP1B* mutated status were taken into consideration. Patients were divided into four groups (bTMB-H/mutant, bTMB-H/co-wild, bTMB-L/mutant and bTMB-L/co-wild). Only four patients fell into the bTMB-H/co-wild group and showed no characteristic mutations compared with other groups (excluded in survival analysis). The PFS and OS were similar in both bTMB-L/co-wild and bTMB-H/mutant groups, and were obviously prolonged compared with the bTMB-L/mutant group (Figure 2F,G). As expected, patients with bTMB-H concomitant *TP53*/*LRP1B* mutation benefited from immunotherapy. It was interesting that patients with *TP53*/*LRP1B* co-wild also benefited from immunotherapy relative to those with *TP53*/*LRP1B* mutant in the bTMB-L group, which deserves to be further explored.

### 3.3. TP53/LRP1B Co-Wild LUSC Had Unequal Mutational Characteristics Compared with Mutant Type

**Figure 3:**
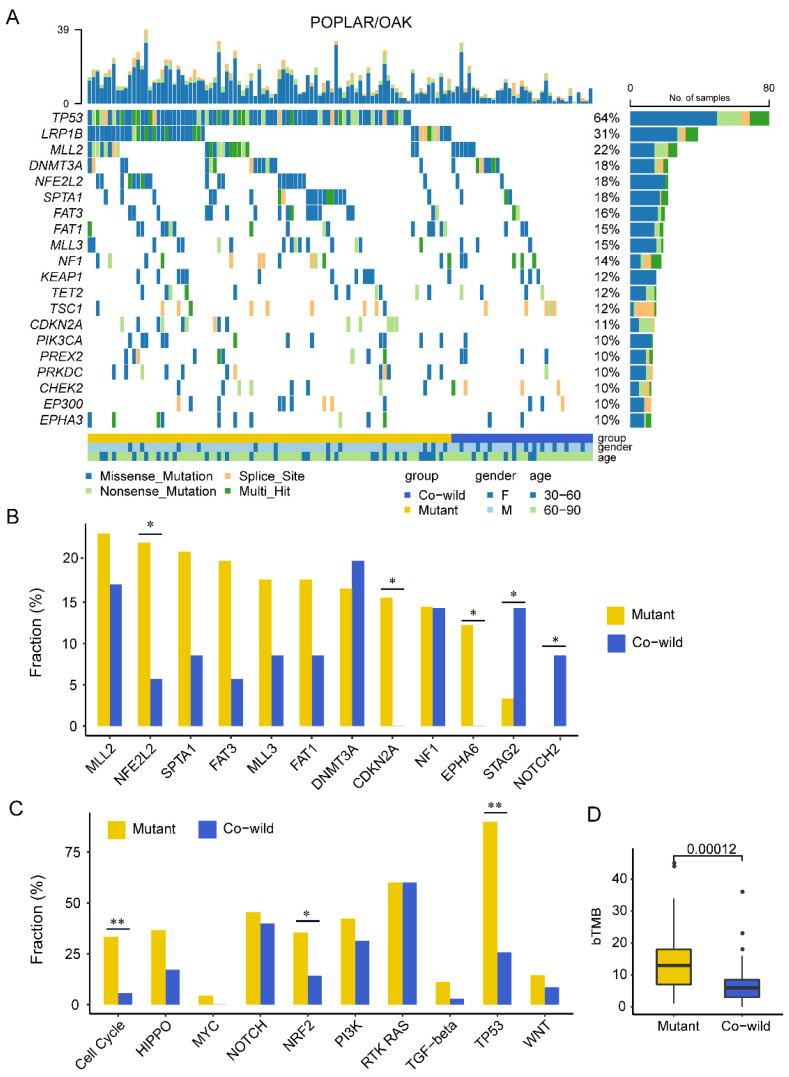
Mutational characteristics of *TP53*/*LRP1B* mutant and co-wild LUSC in POPLAR/OAK cohort. (**A**) The mutational landscapes of *TP53/LRP1B* mutant and co-wild LUSC. (**B**) Comparison of high-frequency and differential genes in two types. (**C**) Comparison of ten oncogenic signaling pathways between *TP53/LRP1B* mutant and co-wild LUSC. (**D**) The bTMB of *TP53/LRP1B* mutant and co-wild LUSC. * *p* < 0.05, and ** *p* < 0.01.

We further focused on exploring the potential mechanism for the immunotherapy benefit of the *TP53*/*LRP1B* co-wild LUSC. We first examined the mutational gene profiles in the POPLAR/OAK cohort. The most common mutated genes except *TP53* and *LRP1B* were *MLL2* (23%), *NFE2L2* (22%), *SPTA1* (21%), *FAT3* (20%), and *MLL3* (18%) in the mutant type (Figure 3A). The co-wild type had frequent *DNMT3A* (20%), MLL2 (17%), EPHA5 (14%), *NF1* (14%), *STAG2* (14%), and *TSC1* (11%) (Figure 3A). The remarkably different genes *NFE2L2*, *CDKN2A*, and *EPHA6* were more frequent in the mutant type, while *STAG2* and *NOTCH2* in the co-wild type (Figure 3B). We analyzed the relationship of these five genes with prognosis and found that *EPHA6* wild type patients had a longer OS compared with the mutant type (Supplementary Figure S6A–J). These results suggest the effect of differential genes between co-wild and mutant types on immunotherapy prognosis was limited.

We next assessed the oncogenic signaling pathway between *TP53*/*LRP1B* co-wild and mutant types. Cell cycle (33% vs. 6%, *p* = 0.001), NRF2 (36% vs. 14%, *p* = 0.028), and TP53 (90% vs. 26%, *p* < 0.001) pathways were more frequently mutated in the mutant type than the co-wild type (Figure 3C). Survival analysis of the wild type and mutant type of these pathways showed that the TP53 pathway mutant was associated with inferior OS (Supplementary Figure S7A–F). We examined the bTMB of two types and found that the mutant type showed a higher bTMB compared with the co-wild type (13 vs. 6 mutations, *p* < 0.001) (Figure 3D). Meanwhile, mutations in any single gene of *LRP1B* and *TP53* were associated with higher bTMB (Supplementary Figure S8A,B), which is consistent with previous reports [34,35,36].

### 3.4. Copy-Number Variation Profile Revealed a Higher Level of Chromosome Stability of TP53/LRP1B Co-Wild LUSC Compared with Mutant LUSC

**Figure 4:**
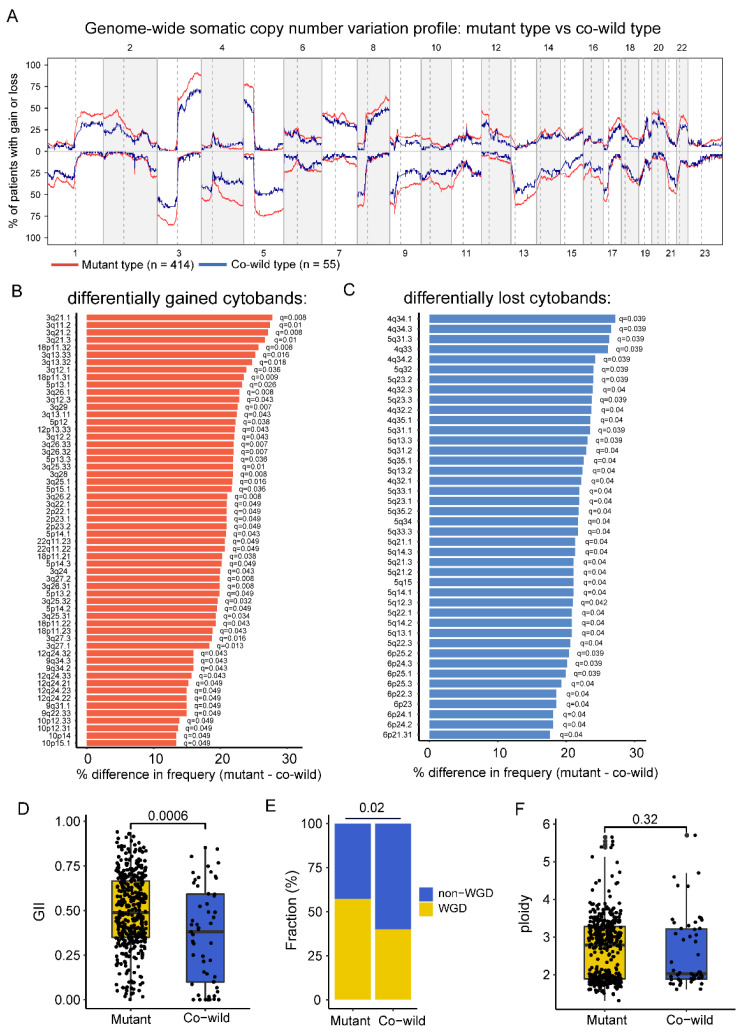
The somatic copy-number alteration profile of *TP53*/*LRP1B* mutant and co-wild LUSC. (**A**) The genome-wide somatic copy number variation profile in *TP53*/*LRP1B* mutant and co-wild LUSC. (**B**) The significant difference in gained cytobands between *TP53/LRP1B* mutant and co-wild LUSC. (**C**) The significant difference in lost cytobands between *TP53/LRP1B* mutant and co-wild LUSC. (**D**–**F**) The GII, WGD, and ploidy difference between *TP53/LRP1B* mutant and co-wild LUSC.

We further explored whether CNV influences the outcome of anti-PD-L1 treatment in *TP53*/*LRP1B* co-wild LUSC. Due to the absence of CNV data in the POPLAR/OAK cohort, we used TCGA CNV data for analysis. The frequency of somatic copy number gain and loss was traced across the whole genome for mutant and co-wild types (Figure 4A). There were 58 gained cytobands and 42 loss cytobands in the mutant type, significantly more than in the co-wild type. No significant cytoband gain or loss, relative to the mutant type, was reported in the co-wild type (Figure 4B,C).

Furthermore, we obtained WGD and ploidy information from a previous study [26], and calculated GII according to copy-number alteration data. The mutant type had a significantly higher GII (0.49 vs. 0.38, *p* < 0.001) and more WGD patients (57% vs. 40%, *p* = 0.020) than the co-wild type (Figure 4D,E). There was no remarkable difference in ploidy between the mutant and co-wild types (2.79 vs. 2.03, *p* = 0.32) (Figure 4F). These results suggested that patients with *TP53*/*LRP1B* mutant LUSC were characterized with a CIN phenotype compared with the co-wild type.

To determine the gene driving the CIN phenotype, we compared the CNV between *TP53*/*LRP1B* co-mutation and *TP53*^mut^/*LRP1B*^wild^ or *TP53^wild^*/*LRP1B^mut^* group and found *TP53* rather than *LRP1B* was associated with the altered cytoband (Supplementary Figure S9). It indicated that *TP53* might drive the copy number phenotype.

### 3.5. TP53/LRP1B Co-Wild LUSC Had Expressional Signatures of Leukocyte Activation and Differentiation

**Figure 5:**
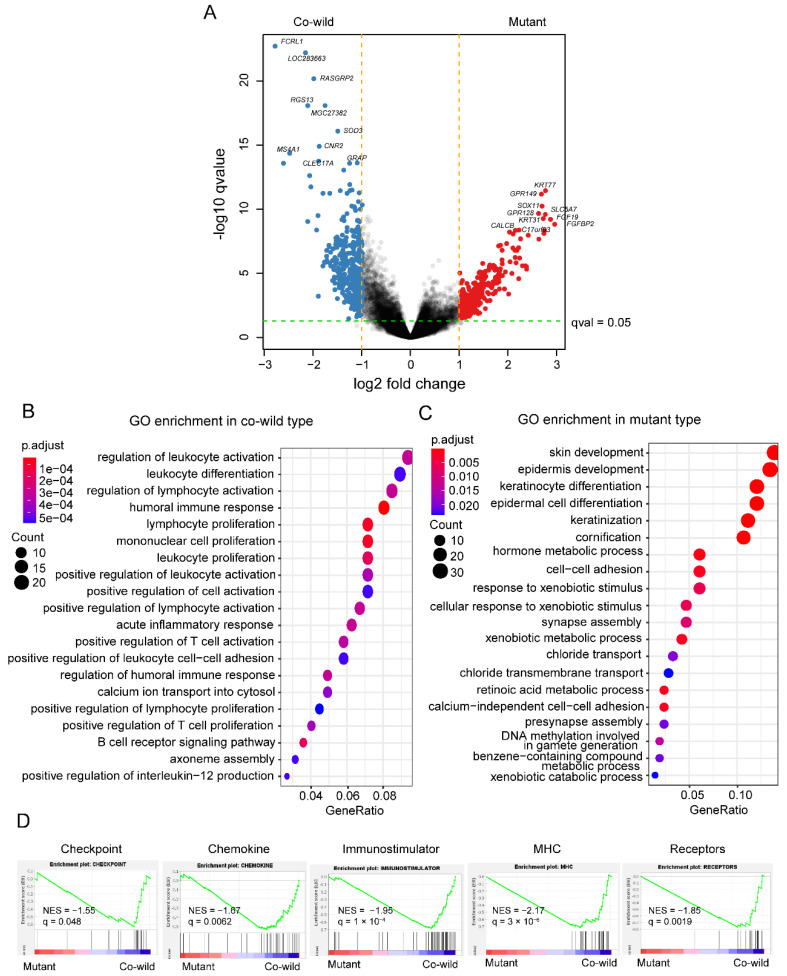
The RNA expression difference between *TP53*/*LRP1B* mutant and co-wild LUSC. (**A**) The results of differentially expressed genes analysis between *TP53/LRP1B* mutant and co-wild LUSC. The labels represent the top 10 genes that significantly changed in the two types. (**B**,**C**) The results of GO enrichment in *TP53/LRP1B* co-wild and mutant co-wild LUSC. (**D**) The results of GSEA analysis base on immune-related gene sets. NES, normalized enrichment score; q, adjusted *p* value.

To investigate the RNA expression difference between mutant and co-wild type, we performed differentially expressed genes (DEG) analysis using RNA expression data of the TCGA cohort. There were 275 genes significantly upregulated in mutant type, while 358 genes in the co-wild type (Figure 5A, Supplementary Tables S5 and S6). *FCRL1*, a B lymphocyte receptor activated costimulatory molecules, exhibited the most significant selective expression in co-wild type compared with the mutant type (log2 fold change = 2.79 versus mutant type, q = 2.02 × 10^−23^) (Figure 5A, Supplementary Table S5). The gene *KRT77* encoding epithelial keratin was most expressed in the mutant type (log2 fold change = 2.76 versus the co-wild type, q = 3.68 × 10^−12^) (Figure 5A, Supplementary Table S6).

GO enrichment was performed on genes with significantly differential expression in the two types respectively. The co-wild type enriched immune regulation terms (e.g., regulation of leukocyte activation, leukocyte differentiation, regulation of lymphocyte activation, humoral immune response, and lymphocyte proliferation) (Figure 5B). The mutant type enriched numerous epidermis-related terms (e.g., skin development, epidermis development, keratinocyte differentiation, and epidermal cell differentiation) (Figure 5C). In addition, gene set enrichment analysis (GSEA) showed immune-related gene sets including checkpoint, chemokine, immunostimulatory, MHC, and receptors were enriched in the co-wild type (Figure 5D). In hallmark gene sets, interferon alpha response, interferon gamma response, and inflammatory response pathway were also enriched in the co-wild type although not significantly (Supplementary Figure S10A). However, cell cycle-related pathways such as E2F target expression, G2M checkpoint, MYC targets version 1, and MYC targets version 2 were expressed more obviously in the mutant type (Supplementary Figure S10B).

### 3.6. TP53/LRP1B Co-Wild LUSC Was Associated with an Activated Immuno-Phenotype

**Figure 6:**
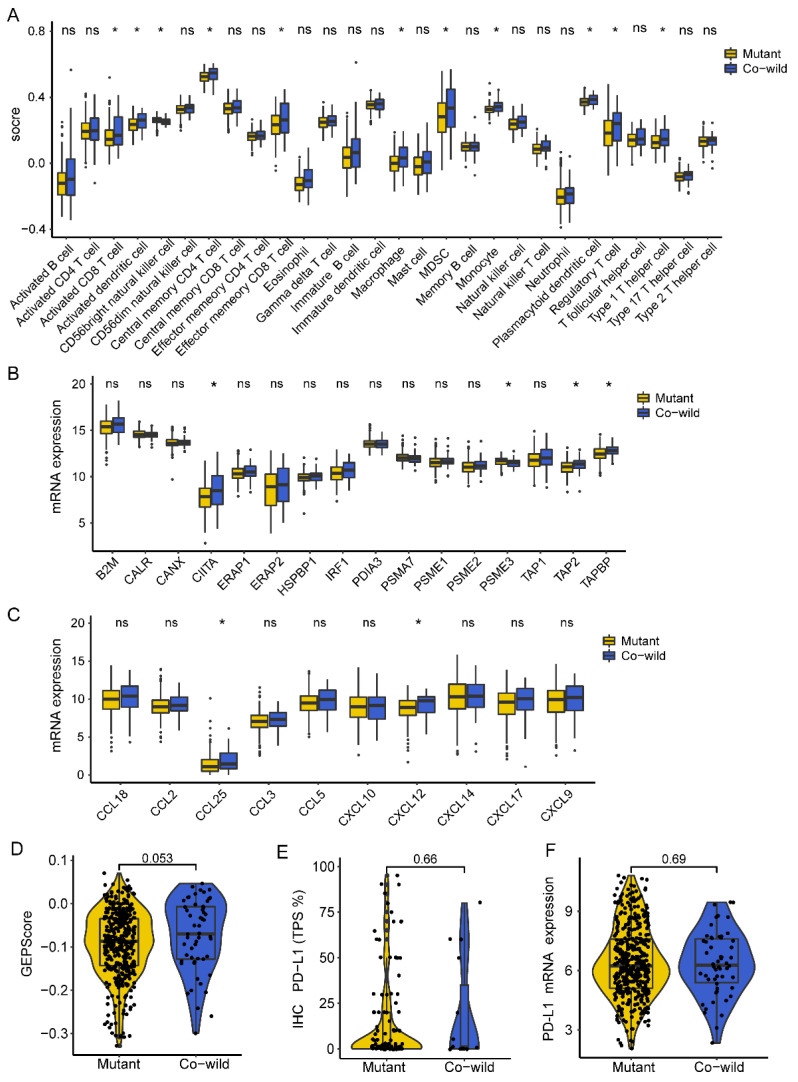
The tumor immune microenvironments of *TP53*/*LRP1B* mutant and co-wild LUSC. (**A**) The results of ssGSEA for *TP53/LRP1B* mutant and co-wild LUSC. (**B**) The difference of antigen presentation related gene expression between *TP53/LRP1B* mutant and co-wild LUSC. (**C**) The difference of chemokine-related genes expression between *TP53/LRP1B* mutant and co-wild LUSC. (**D**) The GEP-score difference between *TP53/LRP1B* mutant and co-wild LUSC. (**E**) The IHC PD-L1 expression difference between *TP53/LRP1B* mutant and co-wild LUSC in the Geneplus cohort. (**F**) The PD-L1 mRNA expression difference between *TP53*/*LRP1B* mutant and co-wild LUSC in the TCGA cohort. * Adjust-*p* < 0.05.

Tumor-infiltrating lymphocytes (TILs) were estimated using TCGA RNA data to characterize the tumor immune microenvironments. We first used the ssGSEA to identify immune cell types. Compared with the mutant type, the co-wild type had a significantly increased proportion of multiple TILs, including activated CD8 T cell, activated dendritic cell (DC), central memory CD4 T cell, effector memory CD8 T cell, macrophage, MDSC, monocyte, plasmacytoid dendritic cell, type 1 T helper cell, and regulatory T cell, while CD56 bright NK cell was rare in the co-wild type (Figure 6A). These results suggested that *TP53*/*LRP1B* alterations took part in an extensively suppressed immune cell infiltration in LUSC.

We next evaluated the expression levels of antigen presentation-related genes and chemokine related genes, and calculated the GEP-score for each sample to assess the immune inflammatory status. The results showed that the antigen presentation-related genes *CIITA*, *TAP2*, and *TAPBP* were highly expressed in the co-wild type (Figure 6B). Two chemokine related genes, *CCL25* and *CXCL12*, were more present in the co-wild than in the mutant type (Figure 6C). The GEP-score of the co-wild type was marginally higher than that of the mutant type, indicating an inflammatory phenotype (Figure 6D). Subsequently, we verified the PD-L1 expression in the Geneplus cohort through IHC, and found that it was not related to *TP53*/*LRP1B* mutation, which is consistent with the results of TCGA PD-L1 mRNA expression (Figure 6E,F).

## 4. Discussion

Immunotherapy can induce a long response in partial patients with LUSC. TMB used as a predictor of immunotherapy efficacy and prognosis remains controversial and limited in LUSC. In this study, we explored the genomic profiles of LUSC in two cohorts of Geneplus and POPLAR/OAK. *TP53* and *LRP1B* were the most frequently mutated genes and correlated with TMB-H. Although TMB alone could not predict immunotherapy efficacy and survival, the combination of TMB and *TP53*/*LRP1B* mutated status could. In patients with *TP53*/*LRP1B* mutation, TMB-H correlated to prolong survival compared with TMB-L. It was interesting that patients with *TP53*/*LRP1B* co-wild were correlated with TMB-L and also improved survival. Further analysis demonstrated *TP53*/*LRP1B* co-wild was associated with a stable genome and higher immune infiltration. *TP53*/*LRP1B* co-wild could act as an ICIs efficacy predictor and prognosis factor in LUSC patients. This novel biomarker could help the classification and identifying the ICIs candidates, which was of great importance for clinical precise treatment.

In the present study, there was high consistency of genomic profiling between Geneplus and POPLAR/OAK cohorts. It indicated that genetic alterations in LUSC were not diverse between races like lung adenocarcinoma (LUAD). *TP53* and *LRP1B* were both the most frequently recurring genes in both cohorts. *TP53* and *LRP1B* mutations were also significantly correlated with TMB-H, which was consistent with previous studies [34,35,36]. We also found *TP53* and *LRP1B* mutations have a synergistic effect in the relationship with TMB. The *TP53*/*LRP1B* co-mutation group was associated with the highest TMB and *TP53*/*LRP1B* co-wild the lowest. Moreover, we observed that the *TP53* mutation was associated with low PD-L1 expression, which was different with previous studies [33,37]. Considering the imbalance of the obtained data, that was, there were 106 patients (84%) with *TP53* mutation but only 21 patients (17%) with high PD-L1; the relationship between *TP53* and PD-L1 needs to be confirmed in a larger sample size.

*TP53* is the most common tumor suppressor gene and plays an important role in regulating cell cycle arrest, apoptosis, and senescence [38]. The inactivation of *TP53* correlated with genome instability and increase of neoantigens, which was associated with improved survival in LUAD patients treated with ICIs [39,40]. A recent study demonstrated that *TP53* mutations were associated with ICI efficacy in advanced NSCLCs [34]. Although PFS was significantly longer in *TP53*-mutated NSCLC (4.5 months), *TP53* mutation status failed to significantly influence PFS in the multivariate analysis. *LRP1B* gene is a member of the low-density lipoprotein receptor family, which participates in clearance of extracellular ligand and extracellular signal transduction [41,42,43]. *LRP1B* is a large gene located on chromosome 2q, containing >91 exons and spanning over 500 kilobases [44]. *LRP1B* was one of the frequently mutated genes in multiple cancer types, such as gastric [41], liver [42], breast [43], and pancreatic cancer [45], and also lung cancer [36]. Due to its large fragment size, it is often overlooked in the analysis of significantly mutated genes. Previous studies reported that *LRP1B* mutations were associated with worse prognosis of hepatocellular carcinoma based on analyses of TCGA and Chinese cohorts [46,47], but a higher TMB and better immunotherapy outcome in NSCLC [36]. Brown et al. proposed the correlation of *LRP1B* alterations and ICI therapy benefit across multiple cancer types including lung cancer [48]. In this study, we also found that almost all of TMB-H patients (only four patients were TMB-H/co-wild, and exhibited no marked mutational characteristics) were concomitant with *TP53*/*LRP1B* mutation, and were associated with favorable survival. However, there was more obvious heterogeneity in the TMB-L group, and patients with *TP53*/*LRP1B* co-wild also benefited from immunotherapy compared with *TP53*/*LRP1B* mutant. This could explain why single TMB could not identify the beneficial population.

To explain why *TP53*/*LRP1B* co-wild was associated with prolonged survival, we conducted subsequent explorations on difference of somatic mutation, CNV, and immune infiltration. *TP53* and *LRP1B* mutant LUSC patients contained an unstable genomic phenotype, which was characterized by a quantity of cytoband-level CNV, including amplification and deletion, higher GII, and a larger WGD proportion. Genomic alterations involving gain or loss of whole chromosomes or structural aberrations are called CIN. Cancers could display various types of CNV, including segmental aneuploidies, focal events, and whole chromosome aneuploidies [49]. An analysis of 5255 TCGA samples revealed that the presence of high-level segmental or whole-chromosome CNVs was correlated with the reduced expression of several genes involved in adaptive immunity and related to cytotoxic CD8+ T cells and NK cells. At the same time, the researchers confirmed that high CNV levels are associated with poor survival using published clinical data on immunotherapy for melanoma patients [50]. Chromosome arm aneuploidies and WGD were reported to shape the tumor evolution and lead to chemotherapeutic drugs resistance [51,52]. Here, increased cell cycle mutations and the activation of E2F and G2M pathways related with CIN and poor prognosis and drug resistance were also determined in LUSC patients with *TP53*/*LRP1B* mutant [38,53]. In short, our results suggested that the increased CIN events caused by the *TP53*/*LRP1B* mutation might be the reason for the poor prognosis of LUSC patients with immunotherapy.

We identified an activated immuno-phenotype in *TP53*/*LRP1B* co-wild LUSC, interpreting the higher response rate and prolonged survival in anti-PD-L1 immunotherapy. In this study, *TP53*/*LRP1B* co-wild LUSC was associated with enhanced levels of activated CD8 T cell, effector memory CD8 T cell, activated dendritic cell, and several chemokines. Accordingly, we speculated that although low TMB might result in low immunogenicity of co-wild type patients, and the stronger dendritic cell activity and the positive effect of certain chemokines induced the infiltration of cytotoxic T cells in these individuals. Several leukocyte activation and differentiation terms identified by GO enrichment provided additional pieces of evidence for the influences of *TP53* and *LRP1B* genes on the immune microenvironment. GSEA showed immune-related gene sets including checkpoint, chemokine, immunostimulatory, MHC, and receptors were enriched in the co-wild type. Moreover, a higher GEP score [28] supported the inflammatory phenotype of *TP53*/*LRP1B* co-wild LUSC. Taken together, these findings indicated that the co-wild type was equipped with an activated immune microenvironment which was more conducive to immunotherapy.

Except for CD8 T cell and activated dendritic cell, *TP53*/*LRP1B* co-wild also had relatively higher levels of suppressor cells in their tumors (i.e., regulatory T cells and MDSCs), which was also found in other reports reflecting the immune activity [54,55]. Regulatory T cells (Treg) inhibit antitumor immunity, maintain immune homeostasis, and correlate with poor prognosis. However, high Treg cell frequency in several cancer types exhibited the opposite result. High Treg cell infiltration was associated with a favorable prognosis in patients with colorectal cancer [56]. Jiae Koh et al. found that high frequencies of circulating Treg cells were correlated with a high response rate, longer PFS and OS, and higher levels of FOXP3+ Treg cells could predict a favorable response to anti-PD-1 immunotherapy in patients with advanced NSCLC [57]. Another study also showed that tumor-infiltrating Tregs were associated with cytotoxic immune responses and prolonged survivalin estrogen receptor-negative breast cancer [58]. Si-Pei Wu found that the frequency of PD-L1 high Tregs was positively correlated with PD-1 high-expressing CD8 in TILs and could act to predict the response to PD-1/PD-L1 blockade immunotherapy [59]. All in above, the mechanism and specific role of immune inhibitory cells such as regulatory T cells and MDSCs are still elusive and deserve further exploration.

We are also aware of some limitations in this study. Due to a few available LUSC immunotherapy cohorts and inadequate materials, the genomic alterations, clinical outcome, CNV, and RNA expression data were collected from different cohorts. Another two major limitations are the lack of validation set and associated biochemical analysis. A prospective study including multi-omics and biochemical analysis could help verify the findings.

The current biomarkers for ICIs such as TMB and PD-L1 are controversial and limited in patients with LUSC. In this study, we found *TP53*/*LRP1B* co-wild LUSC was associated with a better outcome of immunotherapy. *TP53*/*LRP1B* co-wild could act as an ICIs efficacy predictor and prognosis factor in LUSC patients. The reason might be correlated with higher immune infiltration and chromosome stability. Patients with *TP53*/*LRP1B* co-wild genotype might represent a subgroup of the LUSC population. This novel biomarker could help the classification and identification of the ICIs candidates, which is of great importance for precise clinical treatment.

## 5. Conclusions

In summary, *TP53*/*LRP1B* co-wild LUSC conferred an elevated response rate and prolonged survival, which was associated with a chromosome-stable phenotype and activated immune microenvironment.

## Figures and Tables

**Table 1:** Clinical characteristics of the 125 patients with LUSC in the POPLAR/OAK cohort.

Characteristics		All (*n* = 125)	Co-Wild (*n* = 35, 28%)	Mutant (*n* = 90, 72%)	P (Fisher Test)
Age (years)					
	30–60	33 (26%)	6 (17%)	27 (30%)	0.18
	60–90	92 (74%)	29 (83%)	63 (70%)	
Sex					
	Male	103 (82%)	25 (71%)	78 (87%)	0.07
	Female	22 (18%)	10 (29%)	12 (13%)	
Smoking					
	Current	23 (18%)	5 (14%)	18 (20%)	0.22
	Previous	95 (76%)	26 (74%)	69 (77%)	
	Never	7 (6%)	4 (11%)	3 (3%)	
ECOG-PS					
	0	37 (30%)	11 (31%)	26 (29%)	0.83
	1	88 (70%)	24 (69%)	64 (71%)	

## Data Availability

Geneplus cohort data was shown in Supplementary Tables S2 and S3. POPLAR/OAK cohort data was collected from previous study (DOI:10.1038/s41591-018-0134-3). TCGA cohort data was collected from CbioPortal (https://www.cbioportal.org/study/summary?id=lusc_tcga_pan_can_atlas_2018 (accessed on 1 September 2020)).

## Supplementary Materials

The following supporting information can be downloaded at: https://www.mdpi.com/article/10.3390/cancers14143382/s1, Supplementary Figure S1: Comparisons of TMB level between four genosubtypes; Supplementary Figure S2: The mutational characteristics of POPLAR/OAK cohort; Supplementary Figure S3: TMB could not predict the efficacy and survival of immunotherapy in patients with LUSC; Supplementary Figure S4. Effects of *LRP1B* and *TP53* mutation status on PFS (A), OS (B), and efficacy (C). Supplementary Figure S5. The Venn diagram plot of the proportion of *TP53*/*LRP1B* mutant and co-wild type in POPLAR/OAK cohort. Supplementary Figure S6: Survival analysis of five remarkably different genes between *TP53*/*LRP1B* mutant and co-wild LUSC in POPLAR/OAK cohort; Supplementary Figure S7: Comparisons of survival analysis between mutant and wild types in TP53, Cell cycle and NRF2 oncogenic signaling pathway; Supplementary Figure S8: Both *LRP1B* and *TP53* mutation were associated with higher TMB; Supplementary Figure S9: The somatic copy-number alteration profile among *TP53*/*LRP1B* co-mutation, *TP53*^mut^/*LRP1B*^wild^ and *TP53*^wild^/*LRP1B*^mut^ LUSC. Supplementary Figure S10: The results of GSEA analysis base on hallmark gene set. Supplementary Table S1: Detailed List of the 1021-gene-panel in Geneplus cohort; Supplementary Table S2: The mutated genes of 525 patients in Geneplus cohort; Supplementary Table S3: The clinical information and PD-L1 expression data of Geneplus cohort; Supplementary Table S4: Clinical characteristics of the 525 patients in Geneplus cohort; Supplementary Table S5: 358 significantly upregulated genes in *TP53*/*LRP1B* co-wild type. Supplementary Table S6: 275 significantly upregulated genes in *TP53*/*LRP1B* mutant type.

## References

[1] Langer C.J., Obasaju C., Bunn P., Bonomi P., Gandara D., Hirsch F.R., Kim E.S., Natale R.B., Novello S., Paz-Ares L. (2016). Incremental Innovation and Progress in Advanced Squamous Cell Lung Cancer: Current Status and Future Impact of Treatment. J. Thorac. Oncol..

[2] Gandara D.R., Hammerman P.S., Sos M.L., Lara P.N., Hirsch F.R. (2015). Squamous Cell Lung Cancer: From Tumor Genomics to Cancer Therapeutics. Clin. Cancer Res..

[3] Khuder S.A., Mutgi A.B. (2001). Effect of Smoking Cessation on Major Histologic Types of Lung Cancer. Chest.

[4] Herbst R.S., Morgensztern D., Boshoff C. (2018). The biology and management of non-small cell lung cancer. Nature.

[5] Reck M., Rabe K.F. (2017). Precision Diagnosis and Treatment for Advanced Non–Small-Cell Lung Cancer. N. Engl. J. Med..

[6] Brahmer J., Reckamp K.L., Baas P., Crinò L., Eberhardt W.E.E., Poddubskaya E., Antonia S., Pluzanski A., Vokes E.E., Holgado E. (2015). Nivolumab versus Docetaxel in Advanced Squamous-Cell Non–Small-Cell Lung Cancer. N. Engl. J. Med..

[7] Herbst R.S., Baas P., Kim D.-W., Felip E., Pérez-Gracia J.L., Han J.-Y., Molina J., Kim J.-H., Arvis C.D., Ahn M.-J. (2015). Pembrolizumab versus docetaxel for previously treated, PD-L1-positive, advanced non-small-cell lung cancer (KEYNOTE-010): A randomised controlled trial. Lancet.

[8] Paz-Ares L., Luft A., Vicente D., Tafreshi A., Gümüş M., Mazières J., Hermes B., Çay Şenler F., Csőszi T., Fülöp A. (2018). Pembrolizumab plus Chemotherapy for Squamous Non–Small-Cell Lung Cancer. N. Engl. J. Med..

[9] Reck M., Rodríguez-Abreu D., Robinson A.G., Hui R., Csőszi T., Fülöp A., Gottfried M., Peled N., Tafreshi A., Cuffe S. (2016). Pembrolizumab versus Chemotherapy for PD-L1–Positive Non–Small-Cell Lung Cancer. N. Engl. J. Med..

[10] Rittmeyer A., Barlesi F., Waterkamp D., Park K., Ciardiello F., von Pawel J., Gadgeel S.M., Hida T., Kowalski D.M., Dols M.C. (2017). Atezolizumab versus docetaxel in patients with previously treated non-small-cell lung cancer (OAK): A phase 3, open-label, multicentre randomised controlled trial. Lancet.

[11] Lee J.S., Ruppin E. (2019). Multiomics Prediction of Response Rates to Therapies to Inhibit Programmed Cell Death 1 and Programmed Cell Death 1 Ligand 1. JAMA Oncol..

[12] Wang J., Lu S., Yu X., Hu Y., Sun Y., Wang Z., Zhao J., Yu Y., Hu C., Yang K. (2021). RATIONALE-307: Tislelizumab plus chemotherapy versus chemotherapy alone as first-line treatment for advanced squamous NSCLC in patients aged ≥ 65. J. Clin. Oncol..

[13] Mok T.S.K., Wu Y.-L., Kudaba I., Kowalski D.M., Cho B.C., Turna H.Z., Castro G., Srimuninnimit V., Laktionov K.K., Bondarenko I. (2019). Pembrolizumab versus chemotherapy for previously untreated, PD-L1-expressing, locally advanced or metastatic non-small-cell lung cancer (KEYNOTE-042): A randomised, open-label, controlled, phase 3 trial. Lancet.

[14] Hellmann M.D., Ciuleanu T.-E., Pluzanski A., Lee J.S., Otterson G.A., Audigier-Valette C., Minenza E., Linardou H., Burgers S., Salman P. (2018). Nivolumab plus Ipilimumab in Lung Cancer with a High Tumor Mutational Burden. N. Engl. J. Med..

[15] Sacher A.G., Gandhi L. (2016). Biomarkers for the Clinical Use of PD-1/PD-L1 Inhibitors in Non–Small-Cell Lung Cancer. JAMA Oncol..

[16] McGrail D., Pilié P., Rashid N., Voorwerk L., Slagter M., Kok M., Jonasch E., Khasraw M., Heimberger A., Lim B. (2021). High tumor mutation burden fails to predict immune checkpoint blockade response across all cancer types. Ann. Oncol..

[17] Nong J., Gong Y., Guan Y., Yi X., Yi Y., Chang L., Yang L., Lv J., Guo Z., Jia H. (2018). Circulating tumor DNA analysis depicts subclonal architecture and genomic evolution of small cell lung cancer. Nat. Commun..

[18] Li H., Durbin R. (2009). Fast and accurate short read alignment with Burrows—Wheeler transform. Bioinformatics.

[19] Cibulskis K., Lawrence M.S., Carter S.L., Sivachenko A., Jaffe D.B., Sougnez C., Gabriel S.B., Meyerson M.L., Lander E.S., Getz G. (2013). Sensitive detection of somatic point mutations in impure and heterogeneous cancer samples. Nat. Biotechnol..

[20] Yang X., Chu Y., Zhang R., Han Y., Zhang L., Fu Y., Li D., Peng R., Li D., Ding J. (2017). Technical Validation of a Next-Generation Sequencing Assay for Detecting Clinically Relevant Levels of Breast Cancer–Related Single-Nucleotide Variants and Copy Number Variants Using Simulated Cell-Free DNA. J. Mol. Diagn..

[21] McKenna A., Hanna M., Banks E., Sivachenko A., Cibulskis K., Kernytsky A., Garimella K., Altshuler D., Gabriel S., Daly M. (2010). The Genome Analysis Toolkit: A MapReduce framework for analyzing next-generation DNA sequencing data. Genome Res..

[22] Fehrenbacher L., Spira A., Ballinger M., Kowanetz M., Vansteenkiste J., Mazieres J., Park K., Smith D., Artal-Cortes A., Lewanski C. (2016). Atezolizumab versus docetaxel for patients with previously treated non-small-cell lung cancer (POPLAR): A multicentre, open-label, phase 2 randomised controlled trial. Lancet.

[23] Gandara D.R., Paul S.M., Kowanetz M., Schleifman E., Zou W., Li Y., Rittmeyer A., Fehrenbacher L., Otto G., Malboeuf C. (2018). Blood-based tumor mutational burden as a predictor of clinical benefit in non-small-cell lung cancer patients treated with atezolizumab. Nat. Med..

[24] Sanchez-Vega F., Mina M., Armenia J., Chatila W.K., Luna A., La K.C., Dimitriadoy S., Liu D.L., Kantheti H.S., Saghafinia S. (2018). Oncogenic Signaling Pathways in The Cancer Genome Atlas. Cell.

[25] Mermel C.H., Schumacher S.E., Hill B., Meyerson M.L., Beroukhim R., Getz G. (2011). GISTIC2.0 facilitates sensitive and confident localization of the targets of focal somatic copy-number alteration in human cancers. Genome Biol..

[26] Quinton R.J., DiDomizio A., Vittoria M.A., Kotýnková K., Ticas C.J., Patel S., Koga Y., Vakhshoorzadeh J., Hermance N., Kuroda T.S. (2021). Whole-genome doubling confers unique genetic vulnerabilities on tumour cells. Nature.

[27] Liberzon A., Birger C., Thorvaldsdóttir H., Ghandi M., Mesirov J.P., Tamayo P. (2015). The Molecular Signatures Database (MSigDB) hallmark gene set collection. Cell Syst..

[28] Hu J., Yu A., Othmane B., Qiu D., Li H., Li C., Liu P., Ren W., Chen M., Gong G. (2021). Siglec15 shapes a non-inflamed tumor microenvironment and predicts the molecular subtype in bladder cancer. Theranostics.

[29] Charoentong P., Finotello F., Angelova M., Mayer C., Efremova M., Rieder D., Hackl H., Trajanoski Z. (2017). Pan-cancer Immunogenomic Analyses Reveal Genotype-Immunophenotype Relationships and Predictors of Response to Checkpoint Blockade. Cell Rep..

[30] Cristescu R., Mogg R., Ayers M., Albright A., Murphy E., Yearley J., Sher X., Liu X.Q., Lu H., Nebozhyn M. (2018). Pan-tumor genomic biomarkers for PD-1 checkpoint blockade–based immunotherapy. Science.

[31] Arrieta V.A., Cacho-Díaz B., Zhao J., Rabadan R., Chen L., Sonabend A.M. (2018). The possibility of cancer immune editing in gliomas. A critical review. OncoImmunology.

[32] Nagarsheth N., Wicha M.S., Zou W. (2017). Chemokines in the cancer microenvironment and their relevance in cancer immunotherapy. Nat. Rev. Immunol..

[33] Jiang T., Shi J., Dong Z., Hou L., Zhao C., Li X., Mao B., Zhu W., Guo X., Zhang H. (2019). Genomic landscape and its correlations with tumor mutational burden, PD-L1 expression, and immune cells infiltration in Chinese lung squamous cell carcinoma. J. Hematol. Oncol..

[34] Assoun S., Theou-Anton N., Nguenang M., Cazes A., Danel C., Abbar B., Pluvy J., Gounant V., Khalil A., Namour C. (2019). Association of TP53 mutations with response and longer survival under immune checkpoint inhibitors in advanced non-small-cell lung cancer. Lung Cancer.

[35] Lan S., Li H., Liu Y., Ma L., Liu X., Liu Y., Yan S., Cheng Y. (2019). Somatic mutation of LRP1B is associated with tumor mutational burden in patients with lung cancer. Lung Cancer.

[36] Chen H., Chong W., Wu Q., Yao Y., Mao M., Wang X. (2019). Association of LRP1B Mutation with Tumor Mutation Burden and Outcomes in Melanoma and Non-small Cell Lung Cancer Patients Treated With Immune Check-Point Blockades. Front. Immunol..

[37] Xu Y., Li H., Huang Z., Chen K., Yu X., Sheng J., Zhang H.-H., Fan Y. (2020). Predictive values of genomic variation, tumor mutational burden, and PD-L1 expression in advanced lung squamous cell carcinoma treated with immunotherapy. Transl. Lung Cancer Res..

[38] Kent L.N., Leone G. (2019). The broken cycle: E2F dysfunction in cancer. Nat. Cancer.

[39] Dong Z.-Y., Zhong W.-Z., Zhang X.-C., Su J., Xie Z., Liu S.-Y., Tu H.-Y., Chen H.-J., Sun Y.-L., Zhou Q. (2017). Potential Predictive Value of *TP53* and *KRAS* Mutation Status for Response to PD-1 Blockade Immunotherapy in Lung Adenocarcinoma. Clin. Cancer Res..

[40] Lin X., Wang L., Xie X., Qin Y., Xie Z., Ouyang M., Zhou C. (2020). Prognostic Biomarker TP53 Mutations for Immune Checkpoint Blockade Therapy and Its Association with Tumor Microenvironment of Lung Adenocarcinoma. Front. Mol. Biosci..

[41] Takeda H., Rust A.G., Ward J.M., Yew C.C.K., Jenkins N.A., Copeland N.G. (2016). *Sleeping Beauty* transposon mutagenesis identifies genes that cooperate with mutant *Smad4* in gastric cancer development. Proc. Natl. Acad. Sci. USA.

[42] Ding D., Lou X., Hua D., Yu W., Li L., Wang J., Gao F., Zhao N., Ren G., Li L. (2012). Recurrent Targeted Genes of Hepatitis B Virus in the Liver Cancer Genomes Identified by a Next-Generation Sequencing–Based Approach. PLoS Genet..

[43] Asano Y., Takeuchi T., Okubo H., Saigo C., Kito Y., Iwata Y., Futamura M., Yoshida K. (2019). Nuclear localization of LDL receptor-related protein 1B in mammary gland carcinogenesis. J. Mol. Med..

[44] Liu C.-X., Li Y., Obermoeller-McCormick L.M., Schwartz A.L., Bu G. (2001). The Putative Tumor Suppressor LRP1B, a Novel Member of the Low Density Lipoprotein (LDL) Receptor Family, Exhibits Both Overlapping and Distinct Properties with the LDL Receptor-related Protein. J. Biol. Chem..

[45] Cotterchio M., Lowcock E., Bider-Canfield Z., Lemire M., Greenwood C., Gallinger S., Hudson T. (2015). Association between Variants in Atopy-Related Immunologic Candidate Genes and Pancreatic Cancer Risk. PLoS ONE.

[46] Liu F., Hou W., Liang J., Zhu L., Luo C. (2021). LRP1B mutation: A novel independent prognostic factor and a predictive tumor mutation burden in hepatocellular carcinoma. J. Cancer.

[47] Wang L., Yan K., He X., Zhu H., Song J., Chen S., Cai S., Zhao Y., Wang L. (2021). *LRP1B* or *TP53* mutations are associated with higher tumor mutational burden and worse survival in hepatocellular carcinoma. J. Cancer.

[48] Brown L.C., Tucker M.D., Sedhom R., Schwartz E.B., Zhu J., Kao C., Labriola M.K., Gupta R.T., Marin D., Wu Y. (2021). *LRP1B* mutations are associated with favorable outcomes to immune checkpoint inhibitors across multiple cancer types. J. Immunother. Cancer.

[49] Sansregret L., Vanhaesebroeck B., Swanton C. (2018). Determinants and clinical implications of chromosomal instability in cancer. Nat. Rev. Clin. Oncol..

[50] Davoli T., Uno H., Wooten E.C., Elledge S.J. (2017). Tumor aneuploidy correlates with markers of immune evasion and with reduced response to immunotherapy. Science.

[51] Dewhurst S.M., McGranahan N., Burrell R.A., Rowan A.J., Grönroos E., Endesfelder D., Joshi T., Mouradov D., Gibbs P., Ward R.L. (2014). Tolerance of Whole-Genome Doubling Propagates Chromosomal Instability and Accelerates Cancer Genome Evolution. Cancer Discov..

[52] Shukla A., Nguyen T.H.M., Moka S.B., Ellis J.J., Grady J.P., Oey H., Cristino A.S., Khanna K.K., Kroese D.P., Krause L. (2020). Chromosome arm aneuploidies shape tumour evolution and drug response. Nat. Commun..

[53] Ben-David U., Amon A. (2019). Context is everything: Aneuploidy in cancer. Nat. Rev. Genet..

[54] Long J., Wang D., Yang X., Wang A., Lin Y., Zheng M., Zhang H., Sang X., Wang H., Hu K. (2021). Identification of NOTCH4 mutation as a response biomarker for immune checkpoint inhibitor therapy. BMC Med..

[55] Long J., Wang D., Wang A., Chen P., Lin Y., Bian J., Yang X., Zheng M., Zhang H., Zheng Y. (2022). A mutation-based gene set predicts survival benefit after immunotherapy across multiple cancers and reveals the immune response landscape. Genome Med..

[56] Correale P., Rotundo M.S., Del Vecchio M.T., Remondo C., Migali C., Ginanneschi C., Tsang K.Y., Licchetta A., Mannucci S., Loiacono L. (2010). Regulatory (FoxP3+) T-cell Tumor Infiltration Is a Favorable Prognostic Factor in Advanced Colon Cancer Patients Undergoing Chemo or Chemoimmunotherapy. J. Immunother..

[57] Koh J., Hur J.Y., Lee K.Y., Kim M.S., Heo J.Y., Ku B.M., Sun J.-M., Lee S.-H., Ahn J.S., Park K. (2020). Regulatory (FoxP3+) T cells and TGF-β predict the response to anti-PD-1 immunotherapy in patients with non-small cell lung cancer. Sci. Rep..

[58] West N., Kost S.E., Martin S., Milne K., DeLeeuw R.J., Nelson B., Watson P.H. (2012). Tumour-infiltrating FOXP3+ lymphocytes are associated with cytotoxic immune responses and good clinical outcome in oestrogen receptor-negative breast cancer. Br. J. Cancer.

[59] Wu S.-P., Liao R.-Q., Tu H.-Y., Wang W.-J., Dong Z.-Y., Huang S.-M., Guo W.-B., Gou L.-Y., Sun H.-W., Zhang Q. (2017). Stromal PD-L1–Positive Regulatory T cells and PD-1–Positive CD8-Positive T cells Define the Response of Different Subsets of Non–Small Cell Lung Cancer to PD-1/PD-L1 Blockade Immunotherapy. J. Thorac. Oncol..

