# Supplementary material for: TP53 and LRP1B Co-Wild Predicts Improved Survival for Patients with LUSC Receiving Anti-PD-L1 Immunotherapy
Source: Cancers (Basel). 2022 Jul 12;14(14):3382. doi: 10.3390/cancers14143382 (PMC9320428; doi:10.3390/cancers14143382)
Supplement: Supplementary file 1 [file cancers-14-03382-s001.zip › Supplementary Figure--clean version.pdf]

Supplementary Information

TP53 and LRP1B co-wild predicts improved survival for patients with LUSC receiving anti-PD-L1 immunotherapy

Supplementary Figure S1

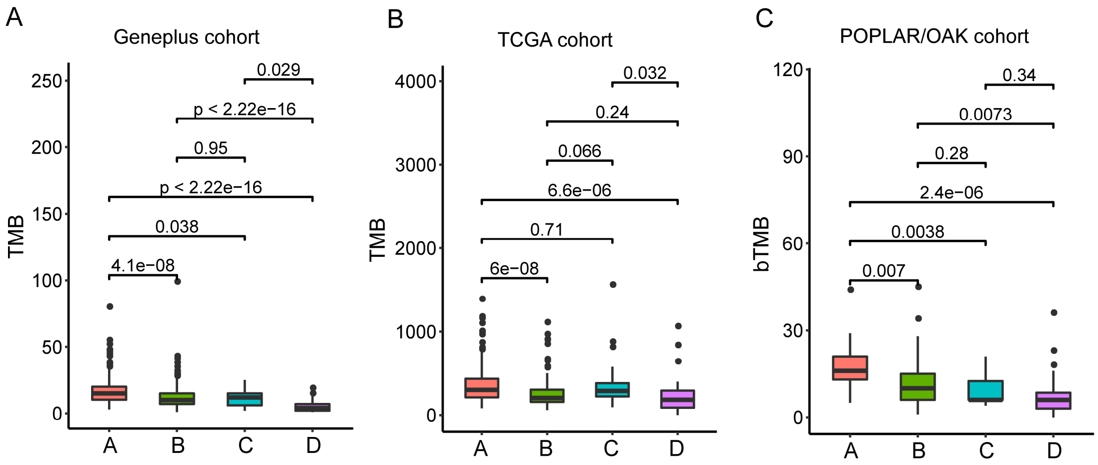

Supplementary Figure S1. Comparisons of TMB level between four genosubtypes. Boxplot (A), (B) and (C) show the TMB levels in Geneplus cohort, TCGA cohort and POPLAR/OAK cohort. A, *TP53*^{mut} and *LRP1B*^{mut}; B, *TP53*^{mut} and *LRP1B*^{wild}; C, *TP53*^{wild} and *LRP1B*^{mut}; D, *TP53*^{wild} and *LRP1B*^{wild}. TMB, tumor mutational burden.

Supplementary Figure S2

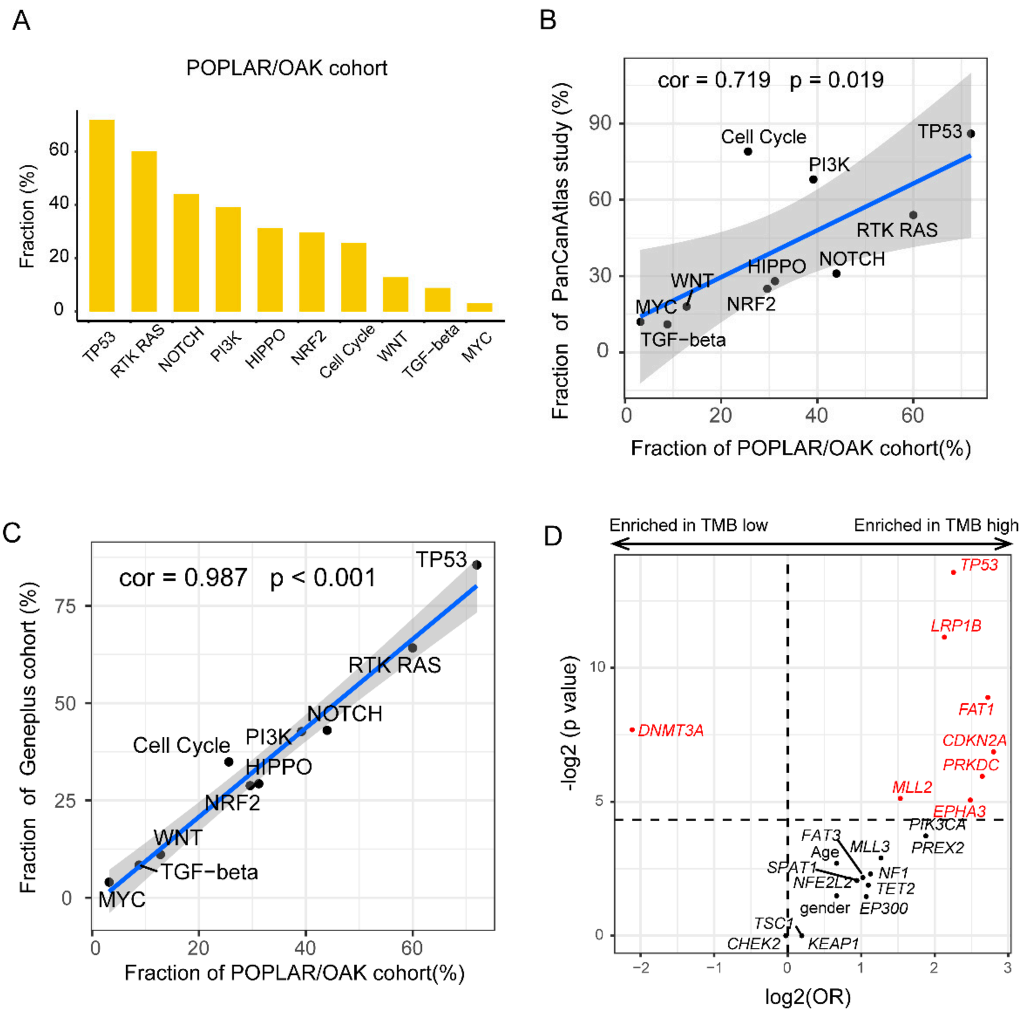

Supplementary Figure S2. The mutational characteristics of POPLAR/OAK cohort. (A) The mutation frequency of ten carcinogenic signaling pathways in POPLAR/OAK cohort. (B)The correlation between the mutation frequency of ten carcinogenic signaling pathways in POPLAR/OAK cohort and PanCanAtlas study. (C) The correlation between the mutation frequency of ten carcinogenic signaling pathways in POPLAR/OAK cohort and Geneplus cohort. (D) The clinical and mutational factors associated with TMB.

Supplementary Figure S3

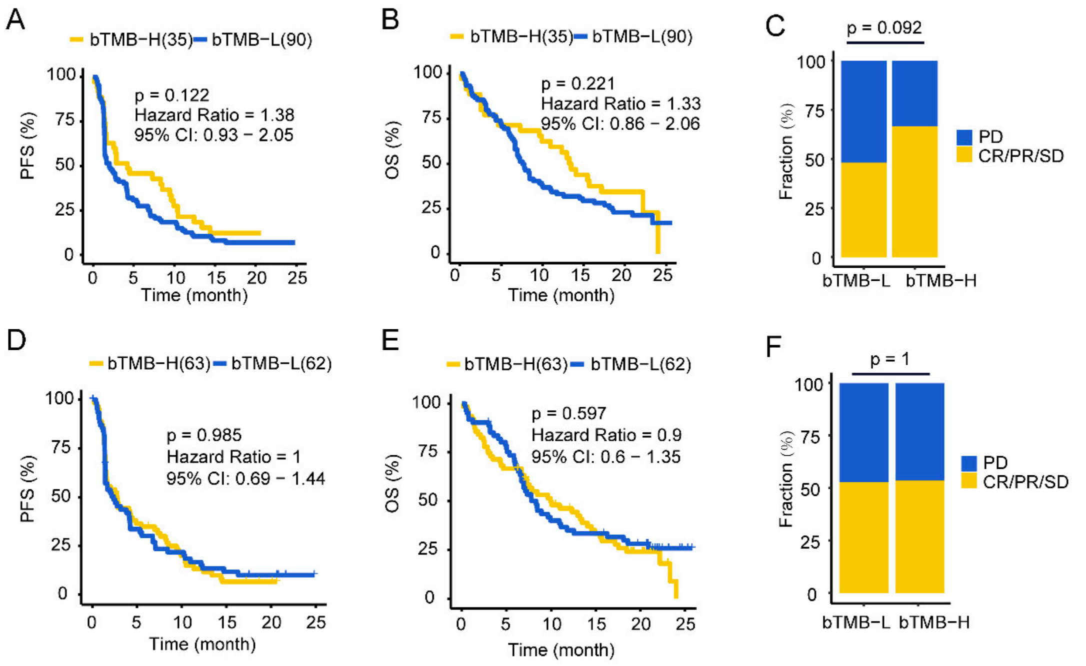

Supplementary Figure S3. TMB could not predict the efficacy and survival of immunotherapy in patients with LUSC.

(A-C) The result of comparison on PFS, OS and DCR between bTMB-H and bTMB-L group using the upper quartile TMB (16 mutations) as cutoff. (D-F) The result of comparison on PFS, OS and DCR between bTMB-H and bTMB-L group using the median TMB (10 mutations) as cutoff. PFS, progression-free survival; OS, overall survival; DCR, disease control rate; bTMB-L, blood tumor mutational burden-low; bTMB-H, blood tumor mutational burden-high.

Supplementary Figure S4

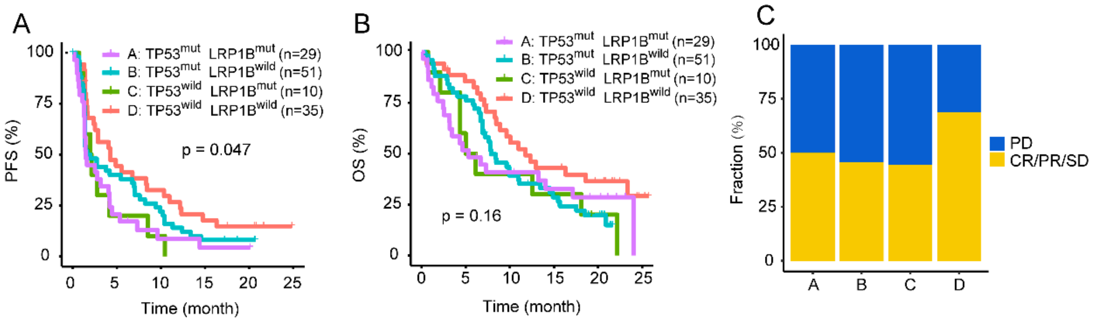

Supplementary Figure S4. Effects of *TP53* and *LRP1B* mutation status on PFS (A), OS (B), and efficacy (C).

Supplementary Figure S5

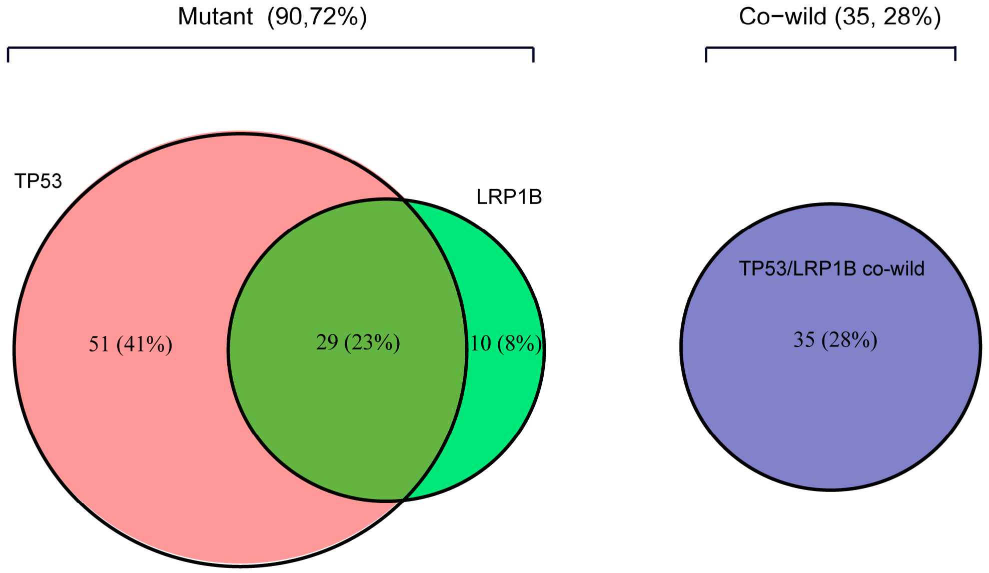

Supplementary Figure S5. The Venn diagram plot of the proportion of *TP53*/*LRP1B* mutant and co-wild type in POPLAR/OAK cohort.

Supplementary Figure S6

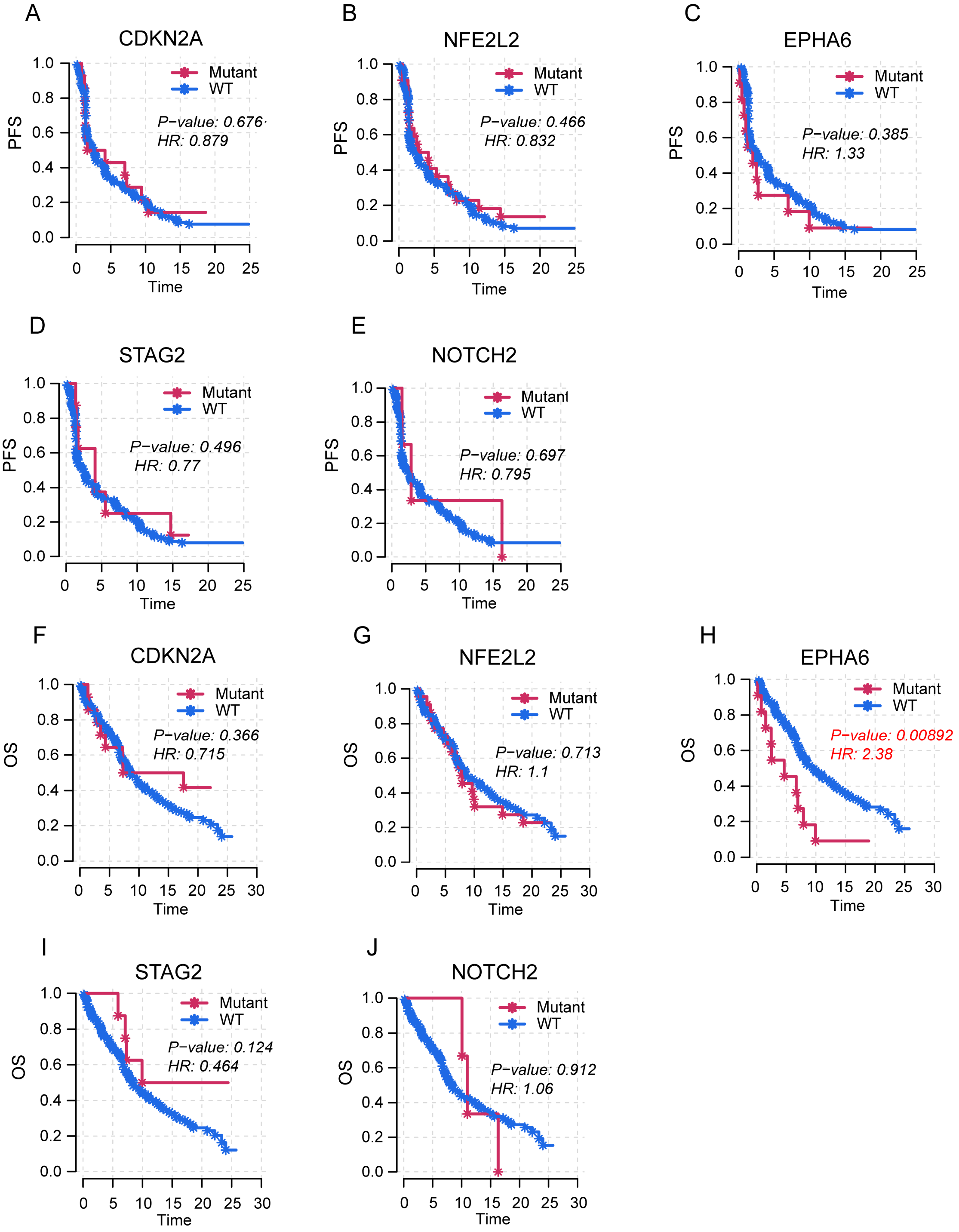

Supplementary Figure S6. Survival analysis of five remarkably different genes between *TP53*/*LRP1B* mutant and co-wild LUSC in POPLAR/OAK cohort.
(A-E) The results of survival analysis on PFS of five remarkably different genes. (F-J) The results of survival analysis on OS of five remarkably different genes.

Supplementary Figure S7

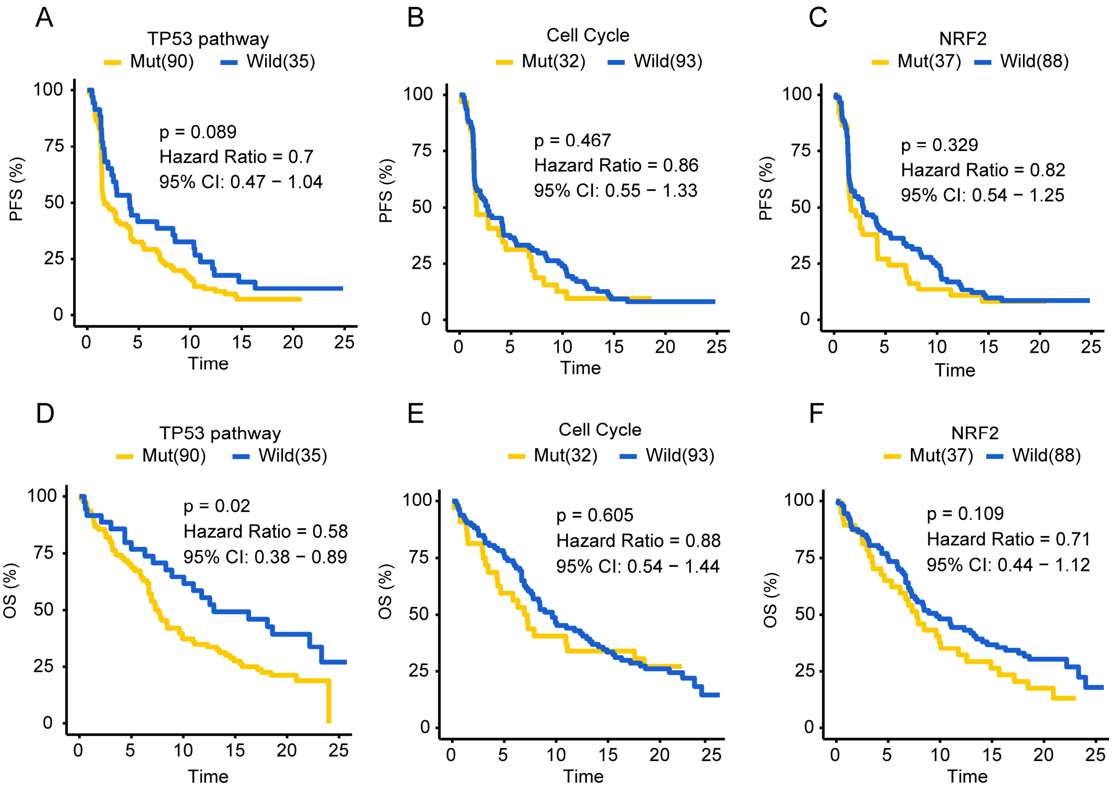

Supplementary Figure S7. Comparisons of survival analysis between mutant and wild types in TP53, Cell cycle and NRF2 oncogenic signaling pathway

(A-C) The results of survival analysis on PFS between mutant type and wild type of three pathways. (D-F) The results of survival analysis on OS between mutant type and wild type of three pathways.

Supplementary Figure S8

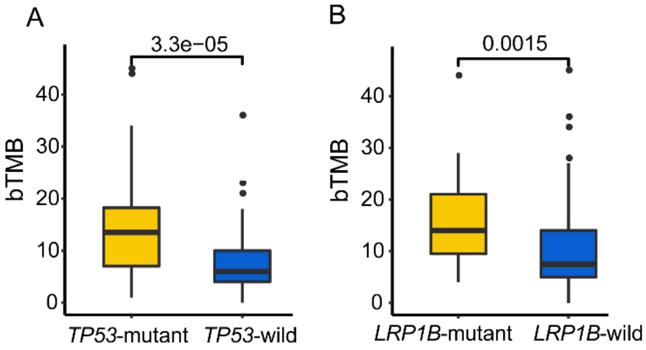

Supplementary Figure S8. Both *LRP1B* and *TP53* mutation were associated with higher TMB.

(A) Comparison of TMB between *TP53*-mutant and *TP53*-wild. (B) Comparison of TMB between *LRP1B*-mutant and *LRP1B*-wild.

Supplementary Figure S9

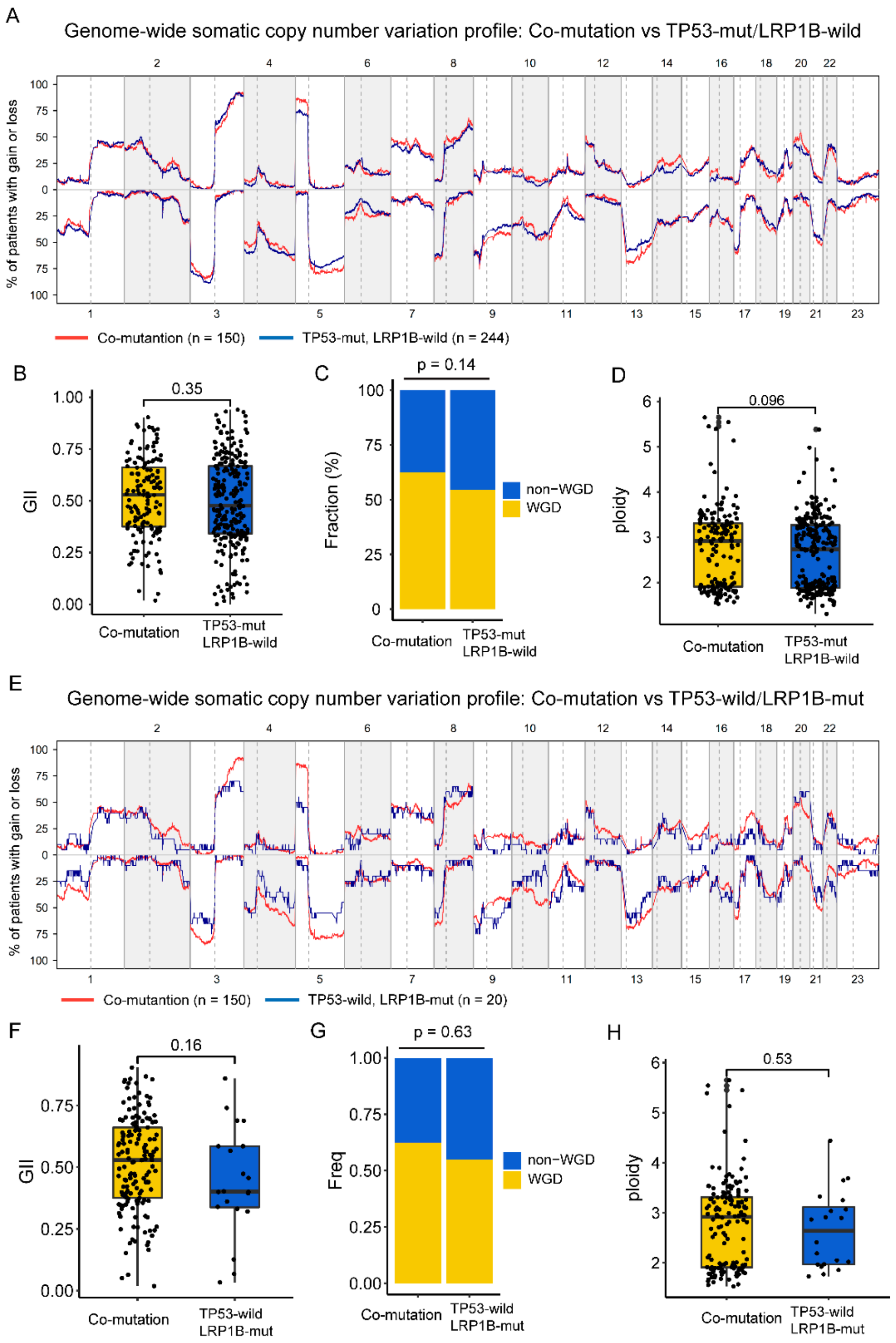

Supplementary Figure S9. The somatic copy-number alteration profile among *TP53*/*LRP1B* co-mutation, *TP53*^{mut}/*LRP1B*^{wild} and *TP53*^{wild}/*LRP1B*^{mut} LUSC. (A) The genome wide somatic copy number variation profile in *TP53/LRP1B* co-mutation and *TP53*^{mut}/*LRP1B*^{wild} LUSC. (B-D) The difference of GII, WGD and ploidy between *TP53/LRP1B* co-mutation and *TP53*^{mut}/*LRP1B*^{wild} LUSC. (E) The genome wide somatic copy number variation profile in *TP53/LRP1B* co-mutation and *TP53*^{wild}/*LRP1B*^{mut} LUSC. (F-H) The difference of GII, WGD and ploidy between *TP53/LRP1B* co-mutation and *TP53*^{wild}/*LRP1B*^{mut} LUSC.

Supplementary Figure S10

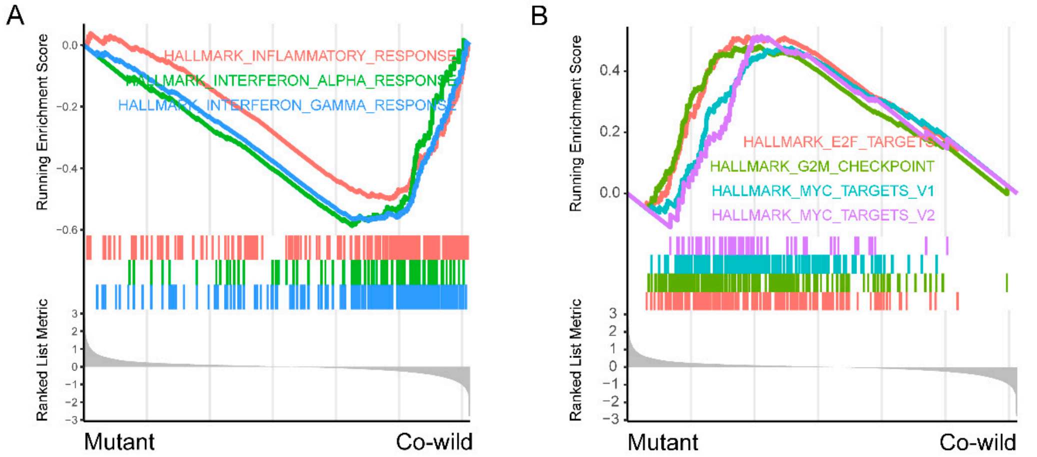

	NES	q value
HALLMARK_INFLAMMATORY_RESPONSE	-1.37	0.308
HALLMARK_INTERFERON_ALPHA_RESPONSE	-1.38	0.310
HALLMARK_INTERFERON_GAMMA_RESPONSE	-1.43	0.391

	NES	q value
HALLMARK_E2F_TARGETS	1.99	0.044
HALLMARK_G2M_CHECKPOINT	2.13	0.034
HALLMARK_MYC_TARGETS_V1	1.99	0.081
HALLMARK_MYC_TARGETS_V2	1.99	0.065

Supplementary Figure S10. The results of GSEA analysis base on hallmark gene set. (A) Three immune-related pathways that tend to be enriched in *TP53/LRP1B* co-wild group. (B) Four pathways that are significantly enriched in the *TP53/LRP1B* mutant group. NES, normalized enrichment score.
