# Supplementary material for: TP53 and LRP1B Co-Wild Predicts Improved Survival for Patients with LUSC Receiving Anti-PD-L1 Immunotherapy
Source: Cancers (Basel). 2022 Jul 12;14(14):3382. doi: 10.3390/cancers14143382 (PMC9320428; doi:10.3390/cancers14143382)
Supplement: Supplementary file 1 [file cancers-14-03382-s001.zip › Supplementary Table S4.pdf]

Supplementary Table S4. Clinical characteristics of the 525 patients in Geneplus cohort.

Characteristics		All(n=525)
Age (years)		
	Median	64
	<30	3 (1%)
	30-60	145 (28%)
	>60	340 (65%)
	NA	37 (7%)
Gender		
	Male	456 (87%)
	Female	69 (13%)
Smoke		
	Smoker	231 (44%)
	Non-smoker	82 (16%)
	NA	212 (40%)
Stage		
	I	4 (1%)
	II	16 (3%)
	III	75 (14%)
	IV	121 (23%)
	NA	309 (59%)
PD-L1		
	0	45 (9%)
	1-49%	60 (11%)
	50-100%	21 (4%)
	NA	399 (76%)

PD-L1, programmed cell death ligand 1; NA, not available.
